# Population Genetic History of *Aristeus antennatus* (Crustacea: Decapoda) in the Western and Central Mediterranean Sea

**DOI:** 10.1371/journal.pone.0117272

**Published:** 2015-03-16

**Authors:** Annamaria Marra, Stefano Mona, Rui M. Sà, Gianfranco D’Onghia, Porzia Maiorano

**Affiliations:** 1 Biology Department, University of Bari, Bari, Italy; 2 Laboratoire Biologie intégrative des populations, Ecole Pratique des Hautes Etudes, Paris, France; 3 School of Biosciences, Cardiff University, Cardiff, United Kingdom; Institute of Biochemistry and Biology, GERMANY

## Abstract

*Aristeus antennatus* is an ecologically and economically important deep-water species in the Mediterranean Sea. In this study we investigated the genetic variability of *A*. *antennatus* sampled from 10 sampling stations in the Western and Central Mediterranean. By comparing our new samples with available data from the Western area, we aim to identify potential genetic stocks of *A*. *antennatus* and to reconstruct its historical demography in the Mediterranean. We analyzed two regions of mitochondrial DNA in 319 individuals, namely COI and 16S. We found two main results: i) the genetic diversity values consistent with previous data within the Mediterranean and the absence of barriers to gene flow within the Mediterranean Sea; ii) a constant long-term effective population size in almost all demes but a strong signature of population expansion in the pooled sample about 50,000 years B.P./ago. We propose two explanation for our results. The first is based on the ecology of *A*. *antennatus*. We suggest the existence of a complex meta-population structured into two layers: a deeper-dwelling stock, not affected by fishing, which preserves the pattern of historical demography; and genetically homogeneous demes inhabiting the fishing grounds. The larval dispersal, adult migration and continuous movements of individuals from “virgin” deeper grounds not affected by fishing to upper fishing areas support an effective ‘rescue effect’ contributing to the recovery of the exploited stocks and explain their genetic homogeneity throughout the Mediterranean Sea. The second is based on the reproduction model of this shrimp: the high variance in offspring production calls for a careful interpretation of the data observed under classical population genetics and Kingman’s coalescent. In both cases, management policies for *A*. *antennatus* will therefore require careful evaluation of the meta-population dynamics of all stocks in the Mediterranean. In the future, it will be particularly relevant to sample the deepest ones directly.

## Introduction

Accurate estimates of genetic variation in marine populations can support adequate strategies for the management of severely exploited fishery resources. Although many of the scientific recommendations for fisheries management are based on outputs of stock assessment models, better integration of genetic information and conventional methods of fisheries stock assessment could significantly improve the quality of management advice [1]. Population genetic analyses can also help to identify localities harbouring high levels of genetic diversity and select marine areas of special interest [2].

Despite the increasing exploitation of the Mediterranean deep-sea resources, considered highly vulnerable to anthropogenic impact [3], the genetic population structure of deep-sea species has been little investigated. Recently, studies on the genetic variability of Mediterranean stocks of the deep-sea shrimps *Aristeus antennatus* and *Aristaeomorpha foliacea*, both representing the main target species of Mediterranean deep-sea trawling, have been carried out, and data on their population genetic structure have been reported mostly in the Western Mediterranean [4,5,6,7] while few studies have also been conducted in the Central-Eastern side of the Mediterranean basin [5,6].

The blue and red shrimp *Aristeus antennatus* (Risso, 1816) is the main target species of deep-sea trawling and one of the most valuable marine resources in the Mediterranean, where thousands of tonnes of these species are landed along the coasts of the western and central basin [8]. *A*. *antennatus* is widely distributed in the Central area of the East Atlantic, in the Mediterranean Sea, with the exception of the North Adriatic Sea [9], in the Indian Ocean, Mozambique and South Africa [10]. In recent years it has also been recorded off the northern coast of Brazil [11].

The blue and red shrimp is an eurybathic species, with a wide depth distribution between 80 and 3300 m [12] although the highest abundance was found between 500 and 700 m and very low densities were detected on the deeper grounds. It is characterized by different migration patterns between the larval and adult stages. According to the few occurrences of the larval stage in superficial waters, therefore far from the deep fishing bottoms, larvae are supposed to shift up across the water column during pelagic development to the surface, where wide and passive displacements due to marine currents occur [13,14]. Afterwards, the post-larvae move to deep-waters for settlement, with the occurrence of small individuals from the upper to lower slope during a sinking phase which covers a wide depth range [12]. Moreover, a female upward displacement during the growth period has been observed, with a progressive exchange of individuals from deeper virgin bottoms, not affected by fishing, to upper fishing grounds [15]. Finally, vertical daily migrations together with horizontal movements have also been described during the adult phase [16].

Although large fluctuations in the *A*. *antennatus* catches according to the season and area have been reported in recent decades [17], a large reduction in its population size and landings due to fishing pressure and physical disturbance have only been documented in the Spanish and Ligurian fisheries [13,18,19]. Moreover, contrasting assessments from underexploitation to high overfishing conditions have been put forward for various Mediterranean stocks of *A*. *antennatus* [8,12,19,20]. Accordingly, the identification of management units seems to be the priority for current research on *A*. *antennatus*, also considering its peculiar life history and its extensive migrations [13].

Previous genetic studies on *A*. *antennatus* indicated the absence of population differentiation in the Western and Central Mediterranean Sea using mtDNA markers [7,21,22]. This pattern has been explained by their relatively recent separation and/or ongoing gene flow within and between these areas, the latter mosty due to pelagic larvae dispersal and adult migrations [4]. However, recent genetic analyses have provided the first evidence of genetic structuring due to hydrographic barriers between the Western and the Eastern Mediterranean [6] while no genetic study has been carried out so far in the Central Mediterranean.

The aim of the present study is: (i) to identify potential genetic stocks of *A*. *antennatus* in the Western and Central Mediterranean and to test for the presence of barriers to gene flow, which may have shaped its genetic variability; (ii) to reconstruct the historical demography of *A*. *antennatus* in the Mediterranean by comparing our new samples from the Western and Central Mediterranean to previous samples collected from Westernmost Mediterranean and the Atlantic Ocean (AO) [6]. The knowledge of the population structure of *A*. *antennatus* in the new study areas as well as a better characterization of its evolutionary history will contribute to the development of the Mediterranean-wide management strategies for this important commercial species.

## Materials and Methods

### Data collection

A total of 319 individuals were collected between 530 and 750 m in depth from 10 sampling stations located in 3 basins of the Western and Central Mediterranean (Central-Southern Tyrrhenian Sea, North-Western Ionian Sea and Southern Adriatic Sea) during the 2010–2011 MEDITS experimental surveys [23].

**Fig 1:**
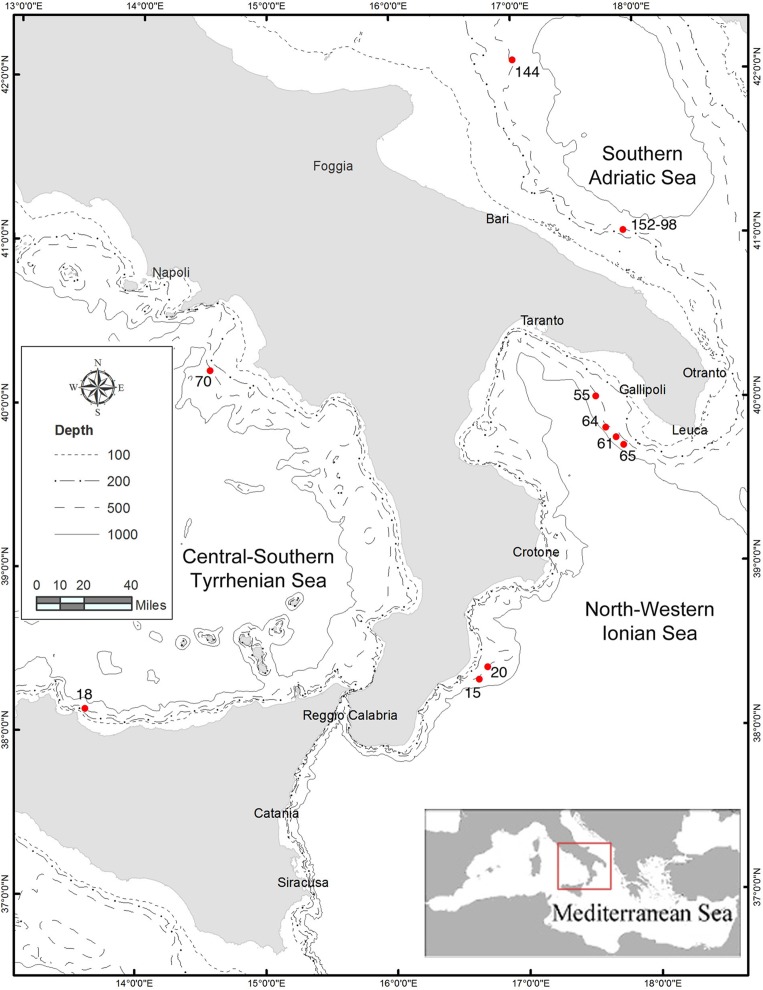
Sampling locations in the Central-Southern Tyrrhenian Sea (Western Mediterranean), North-Western Ionian Sea and Southern Adriatic Sea (Central Mediterranean).

Specific permission was required from the Maritime Authority (Italian Government: Ministero Politiche Agricole Alimentari e Forestali) to carry out the survey at sea and for the locations or activities. The field studies did not involve vulnerable or endangered species. The specific location of the study has been reported in Fig. 1 and Table 1.

**Table 1:** Sampling locations in the Western and Central Mediterranean Sea with indication of the total number of individuals (N) sampled by station.

Geographic area	Station	Depth range (m)	N
**Central-Southern Tyrrhenian Sea**	70	500–600	43
	18	600–700	39
**North-Western Ionian Sea**	20	500–600	36
	15	600–700	48
	55	500–600	44
	64	600–700	24
	61	>700	38
	65	>700	8
**Southern Adriatic Sea**	152–98	500–600	24
	144	600–700	15
**Total**			**319**

The sampling were selected according to the abundance and the preferential distribution of the species in these geographic areas and allocated in three different depth strata: 500–600 m and 600–700 m in all 3 basins, > 700 m only in North-Western Ionian Sea where the species shows the deepest depth distribution. In particular, 2 sampling stations were located in the Central-Southern Tyrrhenian Sea (Western Mediterranean), 6 stations in the North-Western Ionian Sea (Central Mediterranean), and 2 in the Southern Adriatic Sea which represents the northern limit of *A*. *antennatus* distribution in the Adriatic Sea (Central Mediterranean) (Fig. 1, Table 1).

A portion of about 10 mg of muscular tissue was excised from the second pairs of frozen-upon-capture sample pereiopods and was stored in 95% ethanol. DNA was extracted using the Quiagen DNeasy Blood and Tissue Kit. Polymerase chain reactions of the COI and 16S fragments were carried out following the procedures outlined in [7]: 50 μl of reaction volumes containing 5 μl 10X PCR buffer, 1.5 μl MgCl_2_ (50 mM), 5 μl dNTP (2 mM), 1.5 μl of each primer (10 μM), 0.3 μl of 5 units EcoTaq DNA polymerase and 2 μl template. The 16S region was amplified and sequenced using the primers 16SARLpan-T: 5’-TGCCTGTTTATCAAAAACAT-3’and 16SBRHpan: 5’-CCGGTCTGAACTCAAATCATGT-3’ [7]. The COI region was amplified and sequenced using the primers COILAa: 5’-GGTGACCCAGTCCTTTACCA-30 and COIHAa: 5’-GTCTGGATAATCAGAATACCGAC-3’ [7].

The thermal cycling profile began at 94°C for 2 min as a hot start, followed by 35 cycles of 94°C (60 s), 53°C (60 s) and 72°C (60 s), with a final step of 7 min at 72°C for the termination of PCR.

Amplified fragments were screened on 1% agarose gel and the bands were cleaned for sequencing with Exonuclease I and Shrimp Alkaline Phosphatase (SAP) [24]. Purified reaction products were sequenced through capillary electrophoresis at BMR-Genomics Laboratories in Padova, Italy (http://www.bmr-genomics.com). The sequencing primers were the same as those used for the PCR amplifications.

Chromatograms were edited, assembled and aligned using MEGA 5 employing as reference the 16S rDNA and COI sequences of *Aristeus antennatus* obtained by Roldan et al. (2009). Each distinct haplotype has been submitted to GenBank (accession numbers KF768029 to KF768043 for 16S rDNA haplotypes and KF768044 to KF768067 for COI haplotypes).

### Data analysis

Haplotype diversity, nucleotide diversity, neutrality tests (Tajima’s D and Fu’s Fs), pairwise ***ϕ*** *st* and hierarchical analysis of molecular variance (AMOVA) [25] were computed with ARLEQUINv.3.5 [26]. ***ϕ*** *st* takes molecular distance between haplotypes into account and it is therefore more appropriate when dealing with sequence data. The two genes were analyzed separately (COI 500 bp and 16S 447 bp) and concatenated in a single stretch of 947 bp.

AMOVA was applied to partition genetic variance either according to the geographic location or to the depth of the sampled populations. The significance of variance components and ***ϕ***-statistics were assessed by a permutation test with 1,000 bootstrap replicates [25]. The genetic structure of populations and the occurrence of genetic barriers were tested using the SAMOVA program [27]. We ran the simulated annealing algorithm for 10,000 iterations from 5 different random starting points. To find barriers to gene flow we varied *K* (the number of groups) from 2 to 5.

The matrix of pairwise ***ϕ***_*ST*_ was plotted in two dimensions by means of a non-metric multidimensional scaling algorithm [28] as implemented in the *isoMDS* function in R [29].

A median-joining network (MJ) was built using NETWORK 4.5.1.6 [30] with default settings to visualize the evolutionary relationship between haplotypes.

We performed demographic inferences using the Extended Bayesian Skyline [31] coalescent model (EBSP) implemented in BEAST.v.1.7 [32]. We set the clock rate to 1.66% sequence divergence per million years for COI and 0.65% for 16S [7] and generation time to 1 year. We used the Tamura-Nei model of nucleotide substitutionon (TrN, [33]) with a proportion of invariable site (*I*), as selected by means of the Akaike Information Criterion implemented in the program jMODELTEST 0.1.1 [34]. Each analysis was run twice for 100,000,000 iterations with a 10% burn-in and a thinning interval of 10,000. The trace and the effective sample size of all parameters were checked using TRACER.v.1.5 [35].

## Results

A total of 319 concatenated 16S rDNA (447 bp) and COI (500 bp) sequences was obtained from ten sampling station in the Western and Central Mediterranean.

As mtDNA is inherited as a single locus, we preferred to concatenate the two genes but to account for the different in the mutation rate of the two regions we also provided summary statistics for each of them sepatately in the Supplementary Materials (Table A in S1 File) We identify a total of 36 haplotypes in the combined 16S and COI region (Table 2). Analyzed them separately, we found 15 haplotypes in the 16S and 24 in the COI region (Table A in S1 File).

**Table 2:** Diversity measurement for concatenated 16S rDNA and COI sequences (947bp).

Geographic area	Station	N*h*	N*p*	*h*±SD	*π*±SD	TAJIMA'S D	FU'S *Fs*
**Central-Southern Tyrrhenian Sea**	70	8	9	0.340±0.093	0.0008±0.0006	-1.83*	-4.49**
	18	7	8	0.543±0.090	0.0012±0.0009	-1.09	-0.62
	Total	12	13	0.442±0.068	0.0010±0.0007	-1.75*	-7.18**
**North-Western Ionian Sea**	20	6	6	0.427±0.096	0.0010±0.0008	-0.80	-1.35
	15	8	11	0.595±0.055	0.0017±0.0011	-0.92	-1.06
	55	16	18	0.675±0.079	0.0024±0.0015	-1.43*	-7.93**
	64	7	8	0.597±0.107	0.0016±0.0011	-0.85	-1.60
	61	5	5	0.529±0.067	0.0014±0.0010	0:48	0:57
	65	4	5	0.750±0.139	0.0018±0.0013	-0.33	-0.07
	Total	29	26	0.578±0.0358	0.0017±0.0011	-1.75*	-23.21**
**Southern Adriatic Sea**	152–98	6	8	0.543±0.111	0.0014±0.0010	-1.13	-1.00
	144	4	4	0.371±0.153	0.0009±0.0007	-0.92	-0.62
	Total	8	9	0.476±0.094	0.0012±0.0009	-1.29	-2.72*
**Pooled**		**36**	**30**	**0.5361±0.0311**	**0.0015±0.0010**	−1.89**	−35.89**

Number of haplotypes (N*h*); number of polymorphic sites (N*p*); haplotype diversity (*h*); nucleotide diversity (*π*). Tajima’s *D* and Fu’s *Fs* neutrality tests.

* p≤0.05.

**p≤0.005.

Maximum haplotype diversity values was found in the North-Western Ionian Sea, at station 65 (*h*±SD = 0.750±0.139) while the lowest was in the Central-Southern Tyrrhenian, station 70 (*h*±SD = 0.340±0.093). The values for the remaining Mediterranean samples were *h* = 0.371 to 0.675. All summary statistics are presented in Table 2.

**Fig 2:**
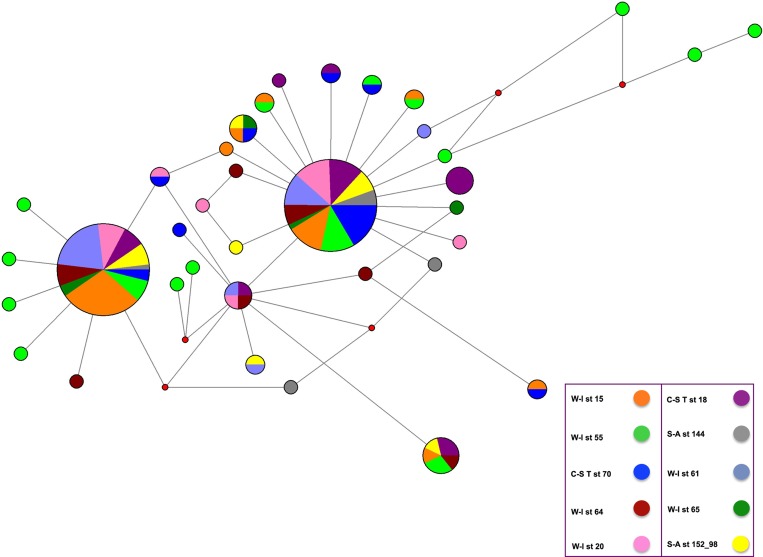
Median-joining network of haplotypes detected for the concatenated 16S rDNA and COI sequences. The area of each circle is proportional to the number of individuals exhibiting that haplotype. Branch length is proportional to the number of mutations occurred. Red dots represent missing or undetected haplotypes. The most frequent haplotypes are shared by individuals from all localities.

We tested for the presence of a significant genetic structure in the Mediterrenean Sea using two different though related approaches: AMOVA and SAMOVA, the latter being able to explicitely take into account geographic positions of the sample. First, we note that that genetic variance was mostly partitioned in the within–population component (98.11%), meaning there are no large difference in alleles distribution among populations (see Fig. 2), even though the fixation index was significantly greater than zero (***ϕ***_*ST*_ = 0.018, P-value < 0.05). Second, when we tested whether geographical areas (Tyrrhenian, Ionian and Adriatic basins) or the fishing depth range (500–600 m, 600–700 m and >700 m), as a good predictor of the genetic variability, we found that ***ϕ***_*CT*_ (which is related to the between group component of the total variance) was not significant in both cases (Table B in S1 File). This means that neither geography nor depth can explain the pattern of genetic variability in our populations. Finally, we searched for groups of populations to maximize the ***ϕ***_*CT*_ using the SAMOVA algorithm. We detected some population partitions with significant (but low) ***ϕ***_*CT*_ values (between 0.043 and 0.046, P-value < 0.05). However, populations within these groups did not display any clear geographic or depth pattern (Table 3), confirming the lack of a clear factor explaing the partition of mtDNA diversity.

**Table 3:** Results of SAMOVA (10,000 iterations) for concatenated sequences (947 bp) of Western and Central Mediterranean.

**Group1:**	**Depth (m)**	**Group2:**	**Depth (m)**		
Southern Adriatic st144	600–700	Western Ionian st 15	600–700		
C-S Tyrrhenian st 18	600–700	Western Ionian st 61	>700		
Western Ionian st 20	500–600				
Western Ionian st 64	600–700				
Western Ionian st 55	500–600				
Western Ionian st 65	>700				
Southern Adriatic st 152_98	500–600				
C-S Tyrrhenian st 70	500–600				
ϕ_CT_ = 0.046*		ϕ_ST_ = 0.044*		ϕ_SC_ = -0.002 *	
**Group1**	**Depth (m)**	**Group2**	**Depth (m)**	**Group3**	**Depth (m)**
Southern Adriatic st 144	600–700	Western Ionian st 15	600–700	Western Ionian st 55	500–600
C-S Tyrrhenian st 18	600–700	Western Ionian st 61	>700		
Western Ionian st 20	500–600	Western Ionian st 64	600–700		
C-S Tyrrhenian st 70	500–600	Western Ionian st 65	>700		
		Southern Adriatic st 152_98	500–600		
ϕ_CT_ = 0.043**		ϕ_ST_ = 0.032*		**ϕ_SC_ = -0.012**	
**Group1**	**Depth (m)**	**Group2**	**Depth (m)**	**Group3**	**Depth (m)**
Southern Adriatic st 144	600–700	Western Ionian st 15	600–700	Western Ionian st 55	500–600
C-S Tyrrhenian st 18	600–700	Western Ionian st 61	>700		
Western Ionian st 20	500–600	Western Ionian st 64	600–700		
C-S Tyrrhenian st 152_98	500–700	Western Ionian st 65	>700		
**Group4**					
C-S Tyrrhenian st 70	500–600				
ϕ_CT_ = 0.046**		ϕ_ST_ = 0.028*		ϕ_SC_ = -0.018 *	
**Group1**	**Depth (m)**	**Group2**	**Depth (m)**	**Group3**	**Depth (m)**
Southern Adriatic st 144	600–700	Western Ionian st 61	>700	Western Ionian st 55	500–600
C-S Tyrrhenian st 18	600–700	Western Ionian st 64	600–700		
Western Ionian st 20	500–600	Western Ionian st 65	>700		
C-S Tyrrhenian st 152_98	500–700				
**Group4**		**Group5**			
C-S Tyrrhenian st 70	500–600	Western Ionian st 15	600–700		
ϕ_CT_ = 0.044**		ϕ_ST_ = 0.024*		ϕ_SC_ = -0.020 *	

We ran the simulated annealing algorithm using different random starting points from two (K = 2) to five groups (K = 5).

*p≤0.05

**p≤0.005. C-S = Central-Southern.

Similarly to the previous analyses, when we included the population samples from Fernandez et al. (2011) (using COI only because the concatenate sequences were not available), the highest percentage of genetic variability (86%) was still present within samples (Table 4). Despite a significant (but low) ***ϕ***_*CT*_ value for K = 5, populations from the same area or depth were located in different groups, suggesting that also in this case no clear geographic or ecological pattern is present (Table 4). This is confirmed by the MDS plot which showed that the studied populations are homogeneous, with the non-Mediterranean Sea sample from the Faro station being the only exception (Fig. A in S1 File). Consistently, the MJ showed a star-like topology, with the most frequent haplotypes shared by individuals from all sites (Fig. 2 and Fig. B in S1 File).

**Table 4:** Results of SAMOVA for five groups (K = 5) using COI sequences.

Locality	Depth (m)	source of variation	d. f.	sum of squares	% of variation	fixation indices	p-value
**Group1**							
Faro (*AO)	na						
**Group2**							
Eastern Ionian (*CM)	na	**Among regions**	4	29.679	12.56	Φ_CT_ = 0.018	0.000
Western Ionian st 65 (CM)	>700						
**Group3**							
Western Ionian st 61 (CM)	>700						
**Group4**							
Western Ionian st 15 (CM)	600–700						
**Group5**							
Alboran (*WM)	na	**Among samples within regions**	15	14.669	1.65	Φ_SC_ = 0.142	0.000
Almeria (*WM)	na						
Soller (*WM)	na						
Cabrera (*WM)	na						
Palamos (*WM)	na						
Lion (*WM)	na						
Genoa (*WM)	na	**Within samples**	806	437.817	85.79	Φ_ST_ = 0.125	0.000
Palermo (*WM)	na						
Western Ionian st 20 (CM)	600–700						
Western Ionian st 55 (CM)	500–600						
C-S Tyrrhenian st 70 (WM)	500–600						
Southern Adriatic st 152_98 (CM)	500–700	**Total**	825	482.166			
Southern Adriatic st 144 (CM)	600–700						
Western Ionian st 64 (CM)	600–700						
C-S Tyrrhenian st 18 (WM)	600–700						

WM: Western Mediterranean, CM: Central Mediterranean, AO: Atlantic Ocean.

* Samples from Fernandez et al. (2010). na: not available.

**Fig 3:**
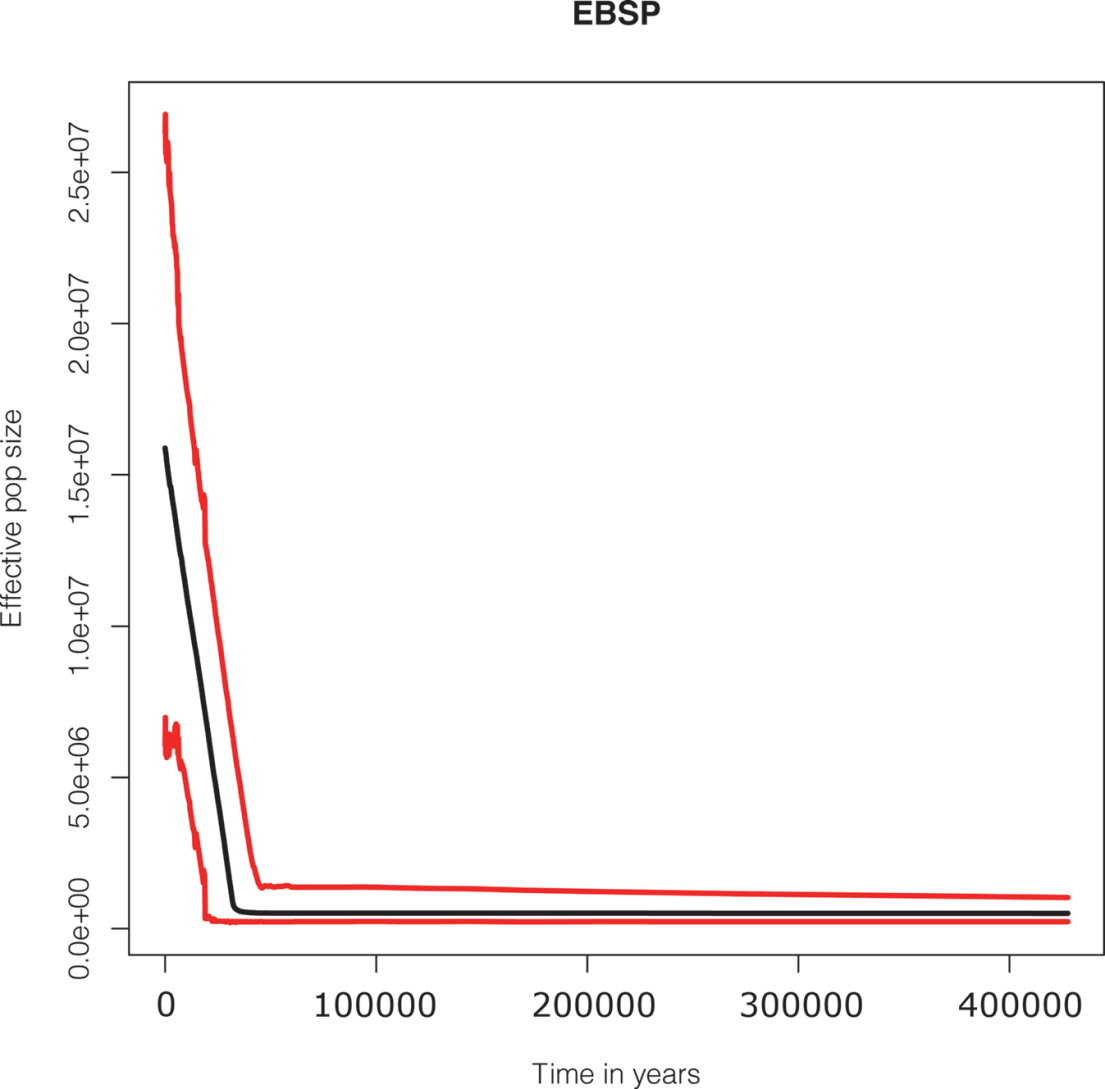
Extended Bayesian Skyline Plot (EBSP) of a concatenated 16S rDNA and COI sequences, Western and Central Mediterranean pooled samples. *X* axis: calendar years. *Y* axis: effective population size. Red lines show the 95% HPD limits; black line the median estimate.

The Extended Bayesian Skyline Plot (EBSP) for the concatenated sequences suggests a strong signature of expansion around 50,000 years B.P., in the pooled Mediterranean, Western-Central Mediterranean and WM samples (Fig. 3, Fig. C in S1 File). Consistently, we obtained significantly negative values of Tajima’s *D* and Fu’*Fs* in the pooled samples, but we detected no departure from equilibrium at the single population level (with the execption of stations 55 and 70) (Table 2).

## Discussion

Genetic diversity values of *A*. *antennatus* throughout the Western and Central Mediterranean were consistent with previous data within the Mediterranean [6,7] but lower than those reported in the nearby Atlantic in Faro, Portugal (*h*±SD = 0.941±0.026; π±SD = 0.0040±0.0004) [6].

The absence of a clear geographic structure suggests that no barriers to gene flow exist within the Mediterranean at the mtDNA level. This confirms the results obtained on the same species by Maggio et. al (2009) and Roldan et al. (2009) with mtDNA, and Cannas et al. (2011) with microsatellite markers, but questions the results presented by Fernandez et al. (2011) who identified the Strait of Sicily as a barrier to gene flow using the same mtDNA markers. However, the latter study inferred the role of the Strait of Sicily in preventing gene flow on the basis of the geographical distribution of genetic variants rather than explicitly testing for it. Here, we have performed rigorous statistical analyses (i.e., SAMOVA) on an extended sample and found no evidence of restricted migrations between the two sides of the Strait.

The only phylogeographic discontinuity we found is represented by the Straits of Gibraltar, which effectively reduces the gene flow between *A*. *antennatus* populations. The topographical and hydrographical characteristics of this Strait would make it difficult for Mediterranean adults and larvae of this species to cross to adjacent Atlantic waters [6]. The Straits are known to prevent gene flow within many other species living between the Atlantic Ocean and the Mediterranean [36] with a consequent large population differentiation across the Atlantic–Mediterranean transition [37,38].

We did not find a signature of a genetic bottleneck, although a reduction in population size due to fishing pressure and physical disturbance has been reported in the WM and an overfishing condition has been observed in various Mediterranean stocks [18,19,20]. Conversely, we found an expansion signature of *A*. *antennatus* around 50,000 years B.P., which may be related to environmental changes which occurred in the Mediterranean Sea. Although different species within the Mediterrenean basin do not show a uniform phylogeographical pattern, three main timings of expansion seem to be common to many of them, namely the Late Pleistocene, the Mid-Pleistocene and the Early Pleistocene [38,39]. The recent paleoclimatic history of the basin is well documented and since the opening of the Straits of Gibraltar, several geological events have influenced the history of the Mediterranean region, most importantly periodic glaciations during the Pleistocene. In particular, the paleoclimatic changes of glacial and interglacial periods occurred during the Middle and Late Pleistocene together with the Mediterranean recolonisation driven by water influx from the adjacent Atlantic Ocean seem to have mostly affected the demographic histories of Mediterranean fauna and designed their genetic structure [40,41]. Indeed, the most recent demographic expansion described for some species, such as the sea urchin *Paracentrotus lividus* [42], the Atlantic Bluefin tuna *Thunnus thynnus* [43] and the sand goby *Pomatoschistus minutus* [44] dated back to between 350,000 and 50,000 years ago and covers almost the same temporal scale we found in *A*. *antennatus*. It is difficult to clearly identify the main ecological forces that can explain the present results, but changes in the trophic conditions with productive environments for many marine species and a general enrichment of the benthic ecosystems at the beginning of the last glacial period (Würm, 70,000–15,000 years ago) could be hypothised [45].

To summarize, the analysis of the mitochondrial genetic variability of *A*. *antennatus* in the Mediterranean Sea leads to three main observations. First: heterozygosity and nucleotidic diversity levels are similar to those described in many decapod crustaceans characterized by an “off shore” life cycle [46]. Second: there are no barriers to gene flow within the Mediterranean, as expected in taxa with high dispersal ability, and the *Fst* correlates neither to geographic distances nor to the depth of the sampling stations. Third: there is generally a constant demography in the single demes with only a few of them showing a recent and slight increase while there is a strong expansion around 50,000 year B.P. in the pooled sample.

Based on these evidences, the present results can be explained considering both the ecological pattern and the specific population genetic of the deep-sea shrimp *A*. *antennatus*.

### Ecological explanation

According to [47] an exchange of individuals from fishing grounds and “virgin” deeper dwelling grounds occurs regularly; in addition, wide horizontal displacements of *A*. *antennatus* have been more recently proposed by [13]. Therefore, we can assume the existence of deeper-dwelling stocks, displaced in areas unsuitable for trawling and consequently not affected by fishing [8], and demes inhabiting the fishing grounds. The deeper-dwelling stock represents the source of a ‘rescue effect’ [48] contributing to the recovery of the upper exploited stocks [22]. Moreover, larval dispersal performed by both vertical ontogenetic migration and horizontal oceanic currents [14], together with the adult migration phenomena and geographical displacements [16,49] increases both vertical and horizontal gene flow as well as connectivity between the sub-populations. Indeed, the blue and red shrimp could be considered “a resource on the move” with horizontal displacements that seem to be more important than vertical ones [13]. These migration patterns and the recolonization of fishing ground by the mobile deeper stocks can explain the absence of population structure at shallower depths and why demes sampled in the same place at almost the same time are as different as any two demes sampled from anywhere in the Mediterranean, as shown by the SAMOVA analysis. This requires that deeper stocks are homogeneous, which would be consistent with what found in several deep sea species of amphipodes, bivalves and gastropods [50,51,52] The demes we sampled are at the shallowest depth of the *A*. *antennatus* distributional range and therefore they are probably recently recolonized units. The shape of the gene genealogy of a deme of a meta-population depends on its effective population size and the migration pattern between demes (*Nm*) [53,54]. For moderate *Nm* values, intra-deme genealogies, particularly for recently occupied demes, will have a mixture of recent and more ancient coalescent events, typical of the constant size demography of an unstructured population [54,55]. This explains our results of the neutrality tests and the Extended Bayesian Skyline Plot at the single population level. Conversely, the gene genealogy of lineages coming from pooled demes is less affected by local coalescence events and it is more sensitive to the historical demography of the whole meta-population [55]. Therefore, our pooled samples provide a picture of the long term effective population size of *A*. *antennatus* in the Mediterranean Sea, apparently not affected by recent fishing pressure or overexploitation conditions. However, we also notice that the exploitation of deep-sea stocks only started in the first few decades of the last century due to the development of the fishing technology in deep waters. In particular, the deep-sea shrimps *A*. *foliacea* and *A*. *antennatus* became the target of deep-water bottom trawl fishing firstly in the Ligurian Sea in the 1930s and in the Catalan Sea in 1940s [56], followed by the other Italian fisheries while they are still almost unexploited in the easternmost part of the Mediterranean [57]. Thus, the growing fishing pressure on deep-sea stocks may still go undetected, especially with mtDNA. A panel of fast mutating loci (i.e., nuclear microsatellites) could be helpful to investigate this issue. Moreover, the absence of analytical results for a two-layer meta-population model would require the use of likelihood free methods such as the approximate Bayesian computation approaches to test this working hypothesis [58]. These methods are computer intensive and would require a large number of independent markers to provide good estimates of the parameter of interest [59], such as the *Nm* values. We therefore performed some indirect inferences using contrasting single deme and pooled deme samples [60] and plan to extend the genetic sampling in the future.

### Population genetic explanation

Species characterized by a large skew in the offspring production as *A*. *antennatus* present more challenge when analysed at the population genetic level. The large variance in the reproductive success determines that gene genealogies are better described by the so called multiple mergers models, where multiple coalescent events per generation can occur, unlike in the Kingman’s n-coalescent [61]. As a consequence, for species characterized by this life trait it is possible to find both shallow genealogies coupled with less genetic diversity than expected on the basis of the census size [62] and “chaotic genetic patchiness”, which can explain the absence of genetic structure as well as the finding of two very close demes showing an higher *Fst* than more distant demes. In these cases, gene genealogies are often expected to be starlike [61] but other factors (like the reduction of number of individuals due to overfishing) can explain while at the single deme level we find only a slight expansion (anyway recent) and not in all stations. Finally we note that pooling lineages for several demes in a meta-population (mimicking a scatter sample sensu Wakeley 1999 [53]) overcome the effect of the skew in offspring production [63]. Therefore, we argue that the finding of an expansion of 50,000 years B.P. is robust to mis-specification of the correct coalescent model.

## Conclusion

We observed a very complex pattern in the genetic variability of *A*. *antennatus*. Ecological and life history traits make the reconstruction of the evolutionary history of the species particularly challenging. We envisioned for the future both to extend the sampling (both geographically and at several depths) and to screen more independent genetic markers in order to achieve a good estimation of the demographic parameters. We also highlight the importance in the next future to extend and apply model based on multiple mergers coalescent to check if they can provide a better interpretation of the data observed. Comparing models based on Kingman’s coalescent and the new extension of coalescent theory [64] would be of crucial importance in the next future. Finally, we note that the role of the deepest virgin stocks is crucial and it should be directly investigated by genetic analyses and accurate sampling despite the difficulties in catching deeper demes. Facing this challenge is fundamental to establish management policies for the protection and conservation of the species.

## Supporting Information

S1 File This file contains Figs. A-C and Tables A and B. Fig. A, MDS Plot based on pairwise F_ST_ values. A) Samples from Western and Central Mediterranean (present study), Western and Central Mediterranean and Atlantic Ocean (from Fernandez et al. 2010); B) Samples from Western and Central Mediterranean Sea (present study). The green rectangles are samples station from Adriatic Sea; the red ones from Ionian Sea; the blue are from Tyrrhenian Sea. The purple rectangles represent samples from Central and Western Mediterranean and the orange one is the Atlantic Ocean sample. Fig. B, Median-joining network of haplotypes detected for 16S rDNA and COI genes from the sampling locations of the Western and Central Mediterranean Sea. The area of each circle is proportional to the number of individuals exhibiting that haplotype. Each line in the network represents one mutational step, and red vertices represent missing or undetected haplotypes. Fig. C, Extended Bayesian Skyline Plot (EBSP) for COI sequences. A) Western and Central Mediterranean (samples from present study); B) Western Mediterranean (samples from present study and from Fernandez et al., 2011); C) All Mediterranean basin (samples from present study and from Fernandez et al., 2011). *X* axis: calendar years. *Y* axis: effective population size (assuming a generation time of one year). The red lines show the 95% HPD limits and the black line is the median estimate. Table A, Diversity measurement for 16S rDNA (447 bp) and COI (500 bp) sequences. In the table are report the summary statistics for each locus gene analyse separately. Number of haplotypes (N*h*); number of polymorphic sites (N*p*); haplotype diversity (*h*); nucleotide diversity (*π*). Tajima’s *D* and Fu’s *Fs* neutrality tests. * p≤0.05, **p≤0.005. Table B, Results of hierarchical analysis of molecular variance (AMOVA) in Central-Eastern Mediterranean. The significance of variance components and *Φ*-statistics was assessed by a permutation test with 1,000 bootstrap replicates. Locality region as in Table 1 and Fig. 1.
(DOC)
