# Correction: Population Genetic History of *Aristeus antennatus* (Crustacea: Decapoda) in the Western and Central Mediterranean Sea

**DOI:** 10.1371/journal.pone.0128609

**Published:** 2015-05-22

**Authors:** Annamaria Marra, Stefano Mona, Rui M. Sà, Gianfranco D’Onghia, Porzia Maiorano

In the Author Contributions section, Porzia Maiorano (PM) should be listed as one of the persons who wrote the paper.
