# Supplementary material for: Population Genetic History of Aristeus antennatus (Crustacea: Decapoda) in the Western and Central Mediterranean Sea
Source: PLoS One. 2015 Mar 16;10(3):e0117272. doi: 10.1371/journal.pone.0117272 (PMC4361500; doi:10.1371/journal.pone.0117272)
Supplement: S1 File — Fig. A, MDS Plot based on pairwise FST values. A) Samples from Western and Central Mediterranean (present study), Western and Central Mediterranean and Atlantic Ocean (from Fernandez et al. 2010); B) Samples from Western and Central Mediterranean Sea (present study). The green rectangles are samples station from Adriatic Sea; the red ones from Ionian Sea; the blue are from Tyrrhenian Sea. The purple rectangles represent samples from Central and Western Mediterranean and the orange one is the Atlantic Ocean sample. Fig. B, Median-joining network of haplotypes detected for 16S rDNA and COI genes from the sampling locations of the Western and Central Mediterranean Sea. The area of each circle is proportional to the number of individuals exhibiting that haplotype. Each line in the network represents one mutational step, and red vertices represent missing or undetected haplotypes. Fig. C, Extended Bayesian Skyline Plot (EBSP) for COI sequences. A) Western and Central Mediterranean (samples from present study); B) Western Mediterranean (samples from present study and from Fernandez et al., 2011); C) All Mediterranean basin (samples from present study and from Fernandez et al., 2011). X axis: calendar years. Y axis: effective population size (assuming a generation time of one year). The red lines show the 95% HPD limits and the black line is the median estimate. Table A, Diversity measurement for 16S rDNA (447 bp) and COI (500 bp) sequences. In the table are report the summary statistics for each locus gene analyse separately. Number of haplotypes (Nh); number of polymorphic sites (Np); haplotype diversity (h); nucleotide diversity (π). Tajima’s D and Fu’s Fs neutrality tests. * p≤0.05, **p≤0.005. Table B, Results of hierarchical analysis of molecular variance (AMOVA) in Central-Eastern Mediterranean. The significance of variance components and Φ-statistics was assessed by a permutation test with 1,000 bootstrap replicates. Locality region as in Table 1 and Fig. 1. (DOC) [file pone.0117272.s001.doc]

**ADDITIONAL FILE**


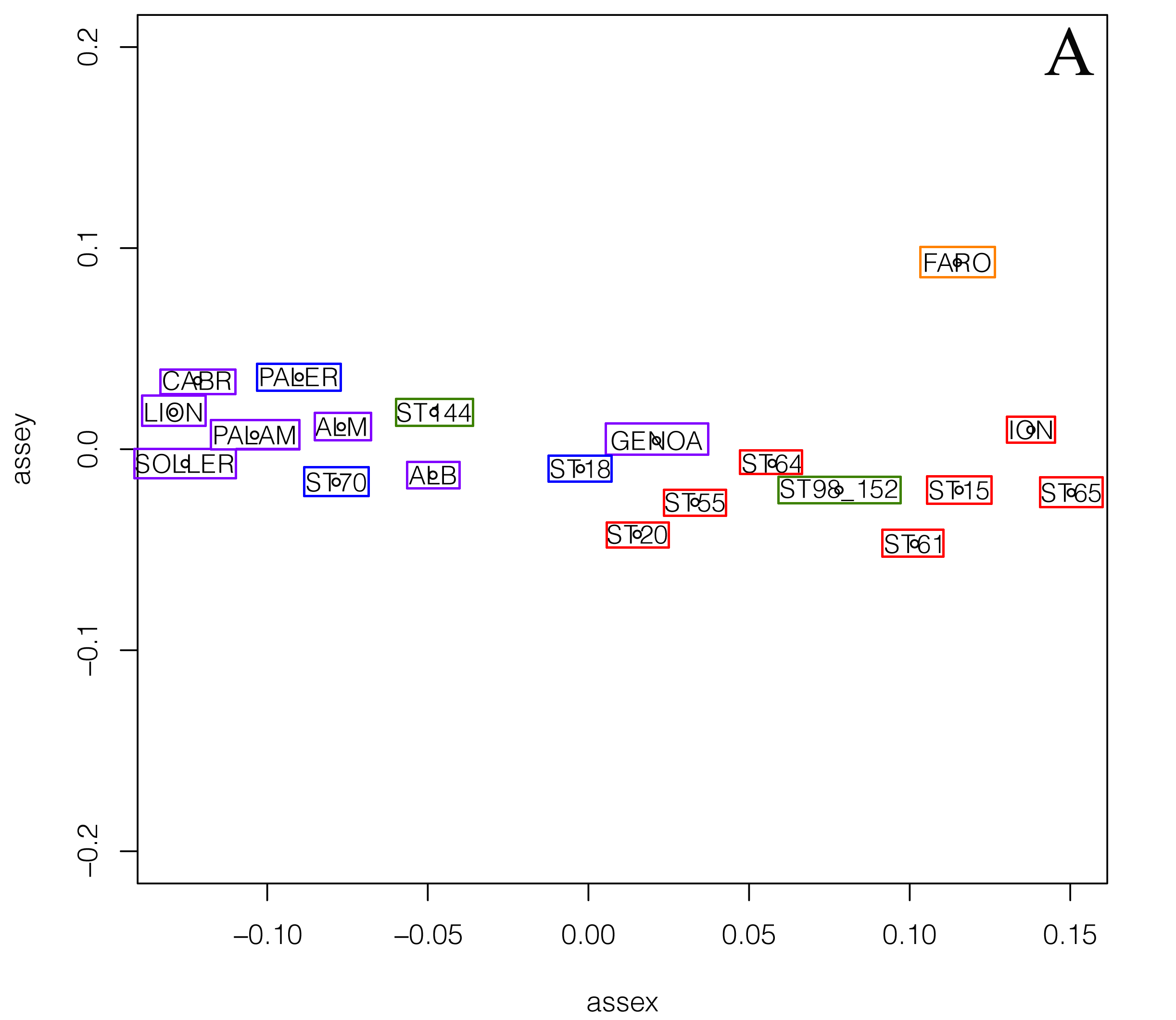

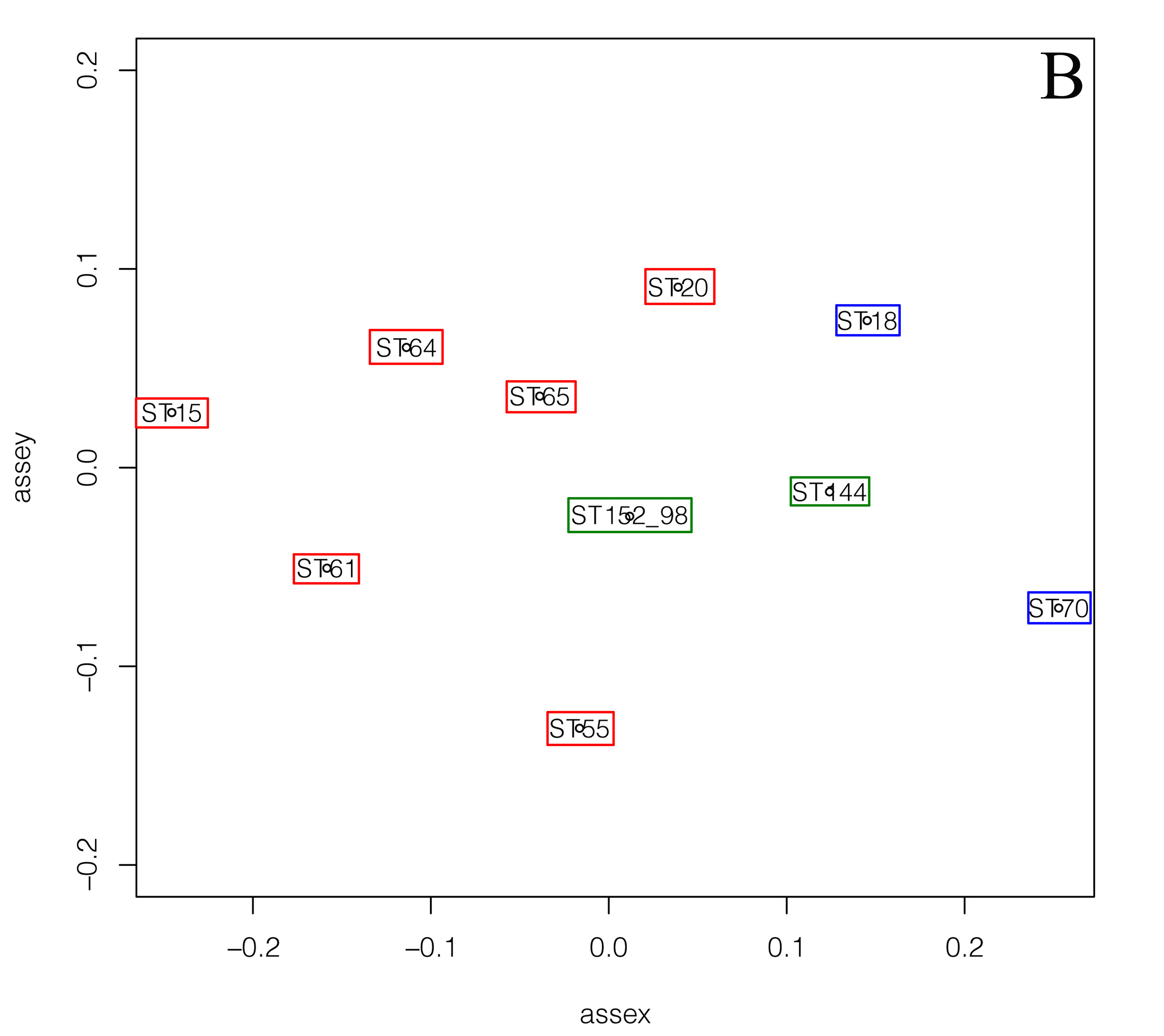


**Figure A – MDS Plot based on pairwise F_ST_ values.** **A)** Samples from Western and Central Mediterranean (present study), Western and Central Mediterranean and Atlantic Ocean (from Fernandez et al. 2010); **B)** Samples from Western and Central Mediterranean Sea (present study).

The green rectangles are samples station from Adriatic Sea; the red ones from Ionian Sea; the blue are from Tyrrhenian Sea.

The purple rectangles represent samples from Central and Western Mediterranean and the orange one is the Atlantic Ocean sample**.**

**Figure B** – **Median-joining network of haplotypes detected for 16S rDNA and COI genes from the sampling locations of the Western and Central Mediterranean Sea.** The area of each circle is proportional to the number of individuals exhibiting that haplotype. Each line in the network represents one mutational step, and red vertices represent missing or undetected haplotypes.

.

**
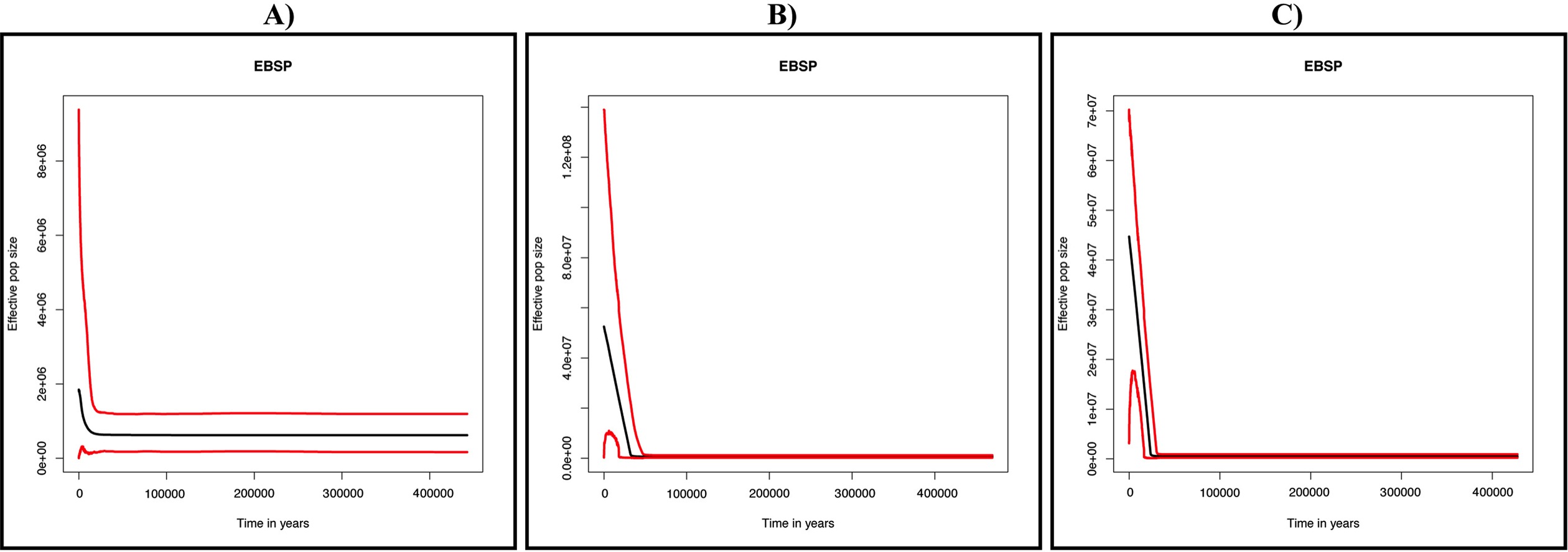
**

**Figure C** –**Extended Bayesian Skyline Plot (EBSP) for COI sequences.** **A)** Western and Central Mediterranean (samples from present study); **B)** Western Mediterranean (samples from present study and from Fernandez et al., 2011); **C)** All Mediterranean basin (samples from present study and from Fernandez et al., 2011).

*X* axis: calendar years. *Y* axis: effective population size (assuming a generation time of one year). The red lines show the 95% HPD limits and the black line is the median estimate.

**TABLE A - Diversity measurement for 16S rDNA (447 bp) and COI (500 bp) sequences.** In the table are report the summary statistics for each locus gene analyse separately. Number of haplotypes (N*h*); number of polymorphic sites (N*p*); haplotype diversity (*h)*; nucleotide diversity **(***π*). Tajima’s *D* and Fu’s *Fs* neutrality tests. * p≤0.05, **p≤0.005.

|  | ***Tot*** | |
| --- | --- | --- |
|  | ***16s*** | ***COI*** |
| **Sample size *N*** | 375 | 376 |
| **No. of polymorphic sites *Np*** | 13 | 17 |
| **N.o of haplotypes *Nh*** | 15 | 24 |
| **Haplotype diversity *h*** | 0.168 | 0.479 |
| **Nucleotide diversity *π*** | 0.00044 | 0.00267 |
| **Tajima’s *D*** | -2.08920* | -1.19888 |
| **Tajima’s D p-value** | < 0.05 | >0.10 |
| **Fu’s *Fs*** | -25.577 | -1.458 |
| **Fu’s Fs p-value** | 0.000 | 0.000 |

**TABLE B** – Results of hierarchical analysis of molecular variance (AMOVA) in Central-Eastern Mediterranean. The significance of variance components and *Φ*-statistics was assessed by a permutation test with 1,000 bootstrap replicates. Locality region as in Table 1 and Fig. 1.

|  | **grouping** | **Source of variation** | **d. f.** | **Sum of squares** | **% of variation** | **Φ-statistic** | **p-value** |
| --- | --- | --- | --- | --- | --- | --- | --- |
| **Depth groups** | **Group1** |  |  |  |  |  |  |
|  | st 20 | Among regions | 2 | 2.794 | 0.40 | Φ_CT_ = 0.004 | 0.397 |
|  | st 55 |  |  |  |  |  |  |
|  | st 70 |  |  |  |  |  |  |
|  | st 152_98 | Among samples | 7 | 7.366 | 1.61 | Φ_SC_ = 0.016 | 0.087 |
|  | **Group2** | within regions |  |  |  |  |  |
|  | st 144 |  |  |  |  |  |  |
|  | st 15 | Within samples | 309 | 217.627 | 97.99 | Φ_ST_ = 0.020 | 0.035 |
|  | st 64 |  |  |  |  |  |  |
|  | st 18 |  |  |  |  |  |  |
|  | **Group3** | Total | 318 | 227.787 |  |  |  |
|  | st 61 |  |  |  |  |  |  |
|  | st 65 |  |  |  |  |  |  |
| **Geographic** | **Group1** |  |  |  |  |  |  |
| **groups** | st 20 | Among regions | 2 | 2.794 | 2.89 | Φ_CT_ = 0.028 | 0.061 |
|  | st 55 |  |  |  |  |  |  |
|  | st 65 |  |  |  |  |  |  |
|  | st 61 | Among samples | 7 | 7.366 | 0.12 | Φ_SC_ = 0.001 | 0.446 |
|  | st 15 | within regions |  |  |  |  |  |
|  | st 64 |  |  |  |  |  |  |
|  | **Group2** |  |  |  |  |  |  |
|  | st 144 | Within samples | 309 | 217.627 | 96.99 | Φ_ST_ = 0.030 | 0.040 |
|  | st 152_98 |  |  |  |  |  |  |
|  | **Group3** | Total | 318 | 227.787 |  |  |  |
|  | st 70 |  |  |  |  |  |  |
|  | st 18 |  |  |  |  |  |  |
